# Prevention of transmission of *Borrelia burgdorferi* sensu lato and *Anaplasma phagocytophilum* by *Ixodes* spp. ticks to dogs treated with the Seresto® collar (imidacloprid 10% + flumethrin 4.5%)

**DOI:** 10.1007/s00436-019-06394-8

**Published:** 2019-11-16

**Authors:** Friederike Krämer, Ricarda Hüsken, Eva Maria Krüdewagen, Katrin Deuster, Byron Blagburn, Reinhard K. Straubinger, Jamie Butler, Volker Fingerle, Sam Charles, Terry Settje, Bettina Schunack, Dorothee Stanneck

**Affiliations:** 1 Institute of Parasitology, Faculty of Veterinary Medicine, Leipzig University, 04103 Leipzig, Germany; 2 TransMIT Gesellschaft für Technologietransfer mbH, 35394 Gießen, Germany; 3 Bayer Animal Health GmbH, 51373 Leverkusen, Germany; 4 Department of Pathobiology, College of Veterinary Medicine, Auburn University, Auburn, AL 36849-5519 USA; 5 Bacteriology and Mycology, Institute for Infectious Diseases and Zoonoses, Department of Veterinary Sciences, LMU Munich, 80539 Munich, Germany; 6 National Reference Centre for Borrelia, Section Infectiology, Bavarian Health and Food Safety Authority (LGL), 85764 Oberschleißheim, Germany; 7 Bayer Animal Health, Shawnee, KS 66216 USA

**Keywords:** Transmission, Canine vector-borne disease (CVBD), Lyme borreliosis, Canine granulocytic anaplasmosis (CGA), Pathogen blocking

## Abstract

The capability of imidacloprid 10% + flumethrin 4.5% (Seresto®) collars to prevent transmission of *Borrelia burgdorferi* sensu lato (*Bb*sl) and *Anaplasma phagocytophilum* (*Ap*) by naturally infected ticks was evaluated in two studies with 44 dogs. In each study, one group served as non-treated control, whereas the other groups were treated with the Seresto® collar. All dogs were exposed to naturally *Bb*sl- and *Ap*-infected hard ticks (*Ixodes ricinus*, *Ixodes scapularis*). In study 1, tick infestation was performed on study day (SD) 63 (2 months post-treatment [p.t.]); in study 2, it was performed on SD 32 (one month p.t.) respectively SD 219 (seven months p.t.). In situ tick counts were performed 2 days after infestation. Tick counts and removals followed 6 (study 1) or 5 days (study 2) later. Blood sampling was performed for the detection of specific *Bb*sl and *Ap* antibodies and, in study 1, for the documentation of *Ap* DNA by PCR. Skin biopsies were examined for *Bb*sl by PCR and culture (only study 1). The efficacy against *Ixodes* spp. was 100% at all time points. In study 1, two of six non-treated dogs became infected with *Bb*sl, and four of six tested positive for *Ap*; none of the treated dogs tested positive for *Bb*sl or *Ap*. In study 2, ten of ten non-treated dogs became infected with *Bb*sl and *Ap*; none of the treated dogs tested positive for *Bb*sl or *Ap*; 100% acaricidal efficacy was shown in both studies. Transmission of *Bb*sl and *Ap* was successfully blocked for up to 7 months.

## Introduction

Vector-borne diseases, especially canine vector-borne diseases (CVBDs), have increasingly become a focus of interest in recent years. The capacity of ticks to transmit pathogens varies widely and often depends on the duration of attachment. Two pathogens, which dogs are frequently confronted with, are *Borrelia burgdorferi* sensu lato (*Bb*sl) and *Anaplasma phagocytophilum* (*Ap*), both transmitted by hard ticks of the genus *Ixodes*.

Lyme borreliosis, a zoonotic disease caused by spirochetes of the *Bb*sl group, affects humans, dogs and other mammalian species (e.g. Chomel 2015). Even though regarding dogs, clinical manifestations are often questionable, not well documented and are not mirrored by the number of seroprevalent dogs in endemic areas (Littman et al. 2018), Lyme borreliosis is reported as the most common vector-borne disease in the USA, Europe and Asia (Wagner et al. 2011). The highly endemic nature of canine *Borrelia* seroprevalence, coupled with its risk to public health in these regions, necessitates tick control in dogs exposed to tick-infested habitats as paramount (Spencer et al. 2003), which is of course also the case in other canine vector-borne diseases with zoonotic character.

Canine granulocytic anaplasmosis (CGA) caused by *Ap*, an obligate, intracytoplasmic coccus that belongs to the family Anaplasmataceae, was reported in the 1980s for the first time in dogs (Madewell and Gribble 1982). Similar to the pathogen of Lyme borreliosis, *Ap* is also zoonotic, so that identical considerations for canine tick control exist.

As Lyme borreliosis and a wide variety of other tick-borne diseases are transmitted via the tick bite, prevention of tick attachment and feeding must be seen as the first obligation of any tick-control agent. Given that no acaricidal compound might be 100% efficacious at preventing tick attachment, as also not requested per guideline (EMA 2016), the final product must be at a minimum 100% efficacious at killing the tick prior to it being able to transmit the pathogen (Spencer et al. 2003).

Hence, knowledge on the transmission times of pathogens—the time needed to transmit pathogens from the vector to the mammalian host after bite/attachment—is especially important for considerations on the capacity of products to inhibit transmission.

Previous studies indicate that transmission time for *Bb*sl is influenced by the *Bb*sl species (Crippa et al. 2002) and may be below 16.7 h (Kahl et al. 1998). The success of transmission of *Bb*sl increases with the duration of attachment for *Ixodes scapularis* (Piesman 1993; des Vignes et al. 2001; Ohnishi et al. 2001) and for *Ixodes ricinus* (Kahl et al. 1998; Crippa et al. 2002). Transmission time for *Ap* generally varies from 24 to ≥ 48 h in small mammals (Katavolos et al. 1998; Hodzic et al. 1998; des Vignes et al. 2001).

The most important product attributes in this context are prevention of biting (an anti-feeding effect) and/or a quick speed of kill to prevent transmission and a residual efficacy to ensure continuous protection. More details on these considerations can be found in Otranto (2018).

Studies focusing on transmission blocking have been conducted for a number of different tick-borne pathogens and tick vectors with a range of different transmission times, using products with different formulations (e.g. collars, spot-ons and orals) and modes of action (contact vs. systemic efficacy) (e.g. Elfassy et al. 2001; Fourie et al. 2013a; Honsberger et al. 2016; Spencer et al. 2003; Taenzler et al. 2015, 2016).

The Seresto® collar (imidacloprid 10% + flumethrin 4.5%) has been commercially available since 2012. The active ingredients have the ability to spread from the collar via the lipid layer of the skin and the hair coat over the surface of the entire treated animal (Stanneck et al. 2012a). The Seresto® collar is highly effective in preventing tick and flea infestations on cats and dogs (Stanneck et al. 2012c) and has also shown to successfully prevent transmission of a range of pathogens including *Ehrlichia canis* (Stanneck and Fourie 2013) and *Babesia vogeli* (Dantas-Torres et al. 2013).

The aim of these two studies reported here was to empirically evaluate the long-term efficacy of the Seresto® collar formulation in preventing the transmission of *Bb*sl and *Ap* to dogs by naturally infected *Ixodes* ticks.

## Study 1 (Germany)

### Materials and methods

#### Study group design

This study was a parallel group design, single centre, randomised, controlled, long-term Good Clinical Practice (GCP) (EMEA 2002) efficacy study involving 14 beagle dogs, conducted at the Animal Centre of Bayer Animal Health, Monheim, Germany. Study design and experimental procedures were approved by the LANUV-Regional authority for nature, environment and consumer protection in North Rhine-Westphalia. Blinding was achieved by separation of function: persons that performed the post-treatment laboratory analysis were different from those that performed group allocation, treatment and sampling.

Fourteen healthy male and female beagle dogs of at least 17 months of age, with a body weight of 9.0 to 12.2 kg and negative for *Bb*sl- and *Ap*-specific antibodies (the same test systems used as for serological testing during the study, see below under “Laboratory procedures”) from a predecessor study, were included in the study. In this former study, two dogs were randomised by number draw prior to further randomisation and received collar treatment in the predecessor study (set as SD 0). They were part of the treatment group of the actual study. The other 12 dogs were ranked according to body weight (highest to lowest), then blocked by two and subsequently randomised by number draw from the six blocks into the following groups, any ties broken by animal ID (highest to lowest):

- Non-treated control group (group 1: *n* = 6)
- Seresto®-treated group, fitted with the collars 2 months prior to tick infestations (2mo-Seresto®, group 2: *n* = 8)

None of the dogs had been treated with an acaricide/insecticide 12 weeks prior to study inclusion. Dogs were acclimated to the study site for at least 14 days and were infested with *I. ricinus* ticks approximately 2 months (study day [SD] 63) after collar placement. Thorough clinical examinations were performed on each study dog pre-inclusion, on SD 0 (treatment day) and then once weekly from SD 1 until SD 181 including the following aspects: body condition, rectal temperature, eyes, cardiovascular system, superficial lymph nodes, ears, respiratory system, gastrointestinal system (oral cavity, anal region, faeces), genitourinary system (external genitalia, urine), skin/hair coat with special attention to the collar application site, behavioural attitude, locomotion/musculature and overall physical condition. Additionally, daily general health observations and measurement of body temperature via a microchip (IPTT-300, BMDS, BioMedic Data Systems, Inc., Seaford, DE, USA) were performed during the course of the study.

#### Dose and administration of the investigational veterinary product

The Seresto® collar (imidacloprid 10% + flumethrin 4.5%) was fitted according to label instructions to the dogs in the treatment group on SD 0.

#### Tick infestation of dogs

On SD 63 (63 days after collar application), approximately 50 adult *I. ricinus* ticks (30 females, 20 males), naturally infected with *Bb*sl and *Ap*, were released onto the sedated study dogs in both groups. Animals were sedated with approximately 0.1 ml/kg body weight ketamine hydrochloride 10% plus approximately 0.1 ml/kg body weight xylazine hydrochloride 2% intramuscularly. Sedation was practiced to allow dispersal and movement of the released ticks into the hair without disturbance. The ticks used for infestation were collected in two infested habitats in Germany by flag dragging (Grafrath and English Garden, Munich, both in Bavaria). The infection rates determined by PCR (as described below under “Culture and molecular screening (*Bb*sl in skin biopsies and ticks)” and “Molecular screening (*Ap* in buffy coat and ticks)”) in two representative samples of at least 100 female ticks of each habitat were 33 and 19.8% for *Bb*sl and 5 and 2% for *Ap* DNA.

#### On animal procedures

##### Tick counting

In situ tick thumb counts by intensive examination and palpation of all body parts without removal of the ticks were carried out on all dogs 48 h after infestation (SD 65) for assessment of acaricidal efficacy. In order to allow sufficient time for potential pathogen transmission, ticks were removed as late as 6 days after infestation (SD 69) when they were again counted and categorised, both according to the World Association for the Advancement of Veterinary Parasitology (W.A.A.V.P.) guideline (Marchiondo et al. 2013). Removed ticks were stored for further pathogen testing (see under “Culture and molecular screening (*Bb*sl in skin biopsies and ticks)” and “Molecular screening (*Ap* in buffy coat and ticks)”).

##### Blood sampling

**Fig. 1:**
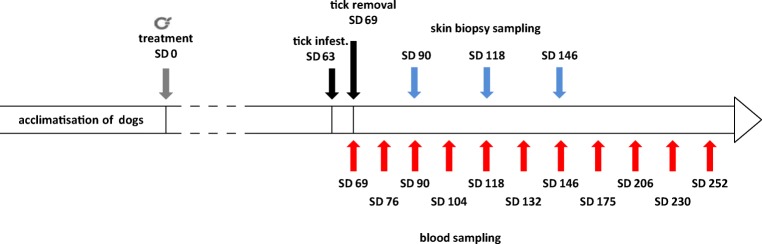
Key study dates of study 1. SD, study day; , collar treatment

Blood sampling for serum and buffy coat collection was performed on all dogs on 6, 13, 27, 41, 55, 69, 83, 112, 143, 167 and 189 days post-tick infestation (i.e. SDs 69, 76, 90, 104, 118, 132, 146, 175, 206, 230 and 252; see Fig. 1 for details). Samples were stored at − 18 °C until analysis.

##### Skin biopsy sampling

Two skin biopsy samples were taken from the area of known tick attachment sites according to the protocol below on the study days as shown in Fig. 1. For the skin biopsy procedure, dogs were sedated as listed above for tick infestation. Skin was shaved and then disinfected thoroughly with betadine 7.5% and thereafter with a commercial available skin disinfectant. The surface of the skin was abraded with a surgical blade and disinfected again. Biopsies were collected with a commercially available 8-mm-diameter punch and skin was closed with a skin stapler. Afterwards, the skin wound was covered with octenidine HCl ointment, and an aluminium wound spray was applied. In cases were no attached ticks were observed, two random sample sites were chosen on the head of the dog, as this is a preferred attachment site of *Ixodes* ticks. Biopsy sample analysis was performed by the National Reference Centre for Borrelia, Section Infectiology, Bavarian Health and Food Safety Authority (LGL), Germany, as described under “Culture and molecular screening (*Bb*sl in skin biopsies and ticks)”.

#### Laboratory procedures

##### Serological screening (*Bb*sl)

Two different serological test systems were used for *Bb*sl antibody detection. A two-step analysis using an automated kinetic enzyme-linked immunosorbent assay (KELA) plus a *Bb*sl immunoblot (abbreviated as IBL) (Borrelia Veterinär plus OspA LINE; Sekisui Virotech GmbH, Rüsselsheim, Germany) and, additionally, a commercially available immunoassay (SNAP® 4Dx®, IDEXX Laboratories, Inc., Westbrook, ME, USA) were performed as per the manufacturers’ standard operating procedures and laboratory procedures. The latter rapid immunoassay was performed and recorded for *Bb*sl antibody detection, even though it was primarily used for serological screening of *Ap* antibody detection (see under “Serological screening (*Ap*)”).

In the two-step test system, the KELA based on *Bb* lysate antigen and recombinant OspA detecting IgG antibody levels (Shin et al. 1993; Barth et al. 2014) was used as the first step. Samples with more than 100 KELA units were considered as positive (above 200 KELA units) and equivocal (between 100 and 200 KELA units), due to cross-reactive antibodies binding to the bacterial lysate used as capture antigens in the screening test. Samples with positive and equivocal KELA results were consecutively analysed qualitatively with the IBL to differentiate IgG reactions to specific antigens (VlsE, OspA, DbpA, OspC [p23], BmpA [p39], p58, p83). Dogs with a negative IBL result, irrespective of a positive or equivocal KELA result, were subsequently considered negative for *Bb*sl antibodies in the two-step test system.

The SNAP® 4Dx® for *Bb*sl detection uses the C6-peptide to detect specific antibodies to the IR6 region of the variable surface protein VlsE [sensitivity 98.8%; specificity 100% (Chandrashekar et al. 2010)].

For the interpretation of the serological results of all three test systems, the following rules for interpretation were used:

**Table:** 

Screening tests (KELA, SNAP® 4Dx®)	Subsequent screening test (IBL)	Interpretation
Positive KELA, negative SNAP® 4Dx®	Positive	Positive
Negative KELA, positive SNAP® 4Dx®	Not performed	Positive
Positive in both tests	Positive	Positive

All blood samples collected were tested for *Bb*sl using SNAP® 4Dx® and KELA, while the IBL was routinely used only at SDs 118 and 175 and additionally in single dogs to clarify positive or equivocal KELA results (SDs 132, 146, 206, 230, 252). All serologic tests were performed at the Institute for Infectious Diseases and Zoonoses, Department of Veterinary Sciences, Faculty of Veterinary Medicine, LMU Munich, Germany.

##### Culture and molecular screening (*Bb*sI in skin biopsies and ticks)

Skin biopsies were cultured in modified Kelly–Pettenkofer (MKP) medium as described by Preac-Mursic et al. (1986).

DNA extraction of the skin biopsy samples and of the removed ticks was performed using QIAGEN DNeasy® Blood & Tissue Kit (QIAGEN GmbH, Hilden, Germany) according to the manufacturer’s instructions. For the detection of bacterial DNA of *Bb*sl, two real-time PCRs were used, targeting *ospA*-gene [13-F (5′-AAT ATT TAT TGG GAA TAG GTC TAA-3′ and 13-R (5′-CAC CAG GCA AAT CTA CTG A-3′)] (Ivacic et al. 2007) and *p41-*gene [Bmp41F (5′-TTG CTT GTG CAA TCA TAG CC-3′) and Bmp41R (5′-GCA AAT CTT GGT GCT TTT CAA-3′), FlaF1 (5′-AGC AAA TTT AGG TGC TTT CCA A-3′) and FlaR1 (5′-GCA ATC ATT GCC ATT GCA GA-3′)] (Venczel et al. 2016). Positive samples were investigated by a differentiating PCR targeting the *hbb* gene [hbb640 forward (5′-GTA AGG AAA TTA GTT TAT GTC TTT *(red640)T-3′) and hbb Bw reverse (5′-TAA GCT CTT CAA AAA AAG CAT CTA-3′)] (Portnoï et al. 2006), which allows species identification by melting curve analysis and/or by an *ospA*-PCR followed by restriction fragment length polymorphism (RFLP) analysis [V1a (forw) primary (5′-GGG AAT AGG TCT AAT ATT AGC-3′), V1b (forw) primary (5′-GGG GAT AGG TCT AAT ATT AGC-3′), V3a (forw) nested (5′-GCC TTA ATA GCA TGT AAG C-3′), V3b (forw) nested (5′-GCC TTA ATA GCA TGC AAG C-3′), R2 (rev) both (5′-CAT AAA TTC TCC TTA TTT TAA AGC-3′), R37 (rev) both (5′-CCT TAT TTT AAA GCG GC-3′)] (Fingerle et al. 2008; Lencakova et al. 2006).

The following Ct values were regarded as cut-off point: In the *p41*-PCR and *ospA*-PCR, a Ct value < 40 was regarded as positive, while in the *hbb*-PCR, a Ct value < 55 was regarded as positive. Samples reactive in *ospA*- and/or *p41*-PCR but negative in *hbb*-PCR and/or *ospA*-RFLP were subjected to sequencing of the *ospA*-fragment for substantiating specificity of the results (Briciu et al. 2014). For this, DNA was sent to QIAGEN for sequencing. Obtained sequences were compared in GenBank.

For the interpretation of the PCR results, the following rules for interpretation were used:

**Table:** 

Screening tests (*ospA, p41*)	Subsequent differentiation (*hbb*, RFLP, sequencing)	Interpretation
Positive in either test	Positive	Positive
Positive in either test	Negative	Negative
Positive in both tests	No further differentiation performed	Positive

##### Serological screening (*Ap*)

All serum samples collected (see Fig. 1) were examined using the SNAP® 4Dx® detecting for *Ap* antibodies to a synthetic peptide from the major surface protein [p44/MSP2; sensitivity 99.1%; specificity 100% (Chandrashekar et al. 2010)]. Cross-reactions with *Anaplasma platys* antibodies have been reported in experimentally infected dogs (Chandrashekar et al. 2010), thus actually stating *Anaplasma* sp. antibodies in the used test. As ticks were confirmed to be *Ap* positive, and *Ap* is the predominant *Anaplasma* species in Northern Europe, positive SNAP® 4Dx® samples were considered to be confirmed as *Ap*-positive samples in the underlying study.

Testing was performed at the Institute for Infectious Diseases and Zoonoses, Department of Veterinary Sciences, Faculty of Veterinary Medicine, LMU Munich, Germany.

##### Molecular screening (*Ap* in buffy coat and ticks)

DNA extraction of the buffy coat samples and of the removed ticks were performed using DNeasy® Blood and Tissue Kit (QIAGEN GmbH, Hilden, Germany) according to the instructions of the manufacturer. To monitor the presence of possibly tick-transmitted *Ap* DNA in the canine buffy coat or in the removed ticks, DNA was analysed by real-time PCR as previously described by Courtney et al. (2004) [ApMSP2f (5′-ATG GAA GGT AGT GTT GGT TAT GGT ATT-3′) and ApMSP2r (5′-TTG GTC TTG AAG CGC TCG TA-3′)]. A Ct value lower than 33 was regarded as positive.

#### Evaluation of efficacy

##### Acaricidal efficacy

To determine the acaricidal efficacy of the treatment, counted ticks were categorised and efficacy was calculated according to the W.A.A.V.P. guideline by Marchiondo et al. (2013). As per this guideline, percentage reduction in tick counts was calculated using the following formula:

$$ \mathrm{Efficacy}\ \left(\%\right)=100\ \mathrm{x}\ \left(\mathrm{Tc}-\mathrm{Tt}\right)/\mathrm{Tc} $$

wherein Tc and Tt were the tick count means of the non-treated control and the treated group, respectively. Effectiveness was calculated based on geometric and arithmetic group means.

##### Pathogen blocking efficacy

The percentage blocking efficacy for the treatment group was calculated for each of the pathogens as follows:

$$ \mathrm{Efficacy}\ \left(\%\right)=100\ \mathrm{x}\ \left(\mathrm{Tc}-\mathrm{Tt}\right)/\mathrm{Tc} $$

Tc: Total number of infected dogs in the non-treated control group
Tt: Total number of infected dogs in the treatment group

#### Statistical analysis

Statistical test analysis was done with the non-parametric Wilcoxon–Mann–Whitney *U* test and the unconditional exact Röhmel–Mansmann test, an exact non-parametric analysis for 2 × 2 tables (two-sided, alpha = 0.05), for the group comparisons: treatment versus control. Target parameters were tick counts at 48 h *post infestationem* (p.i.) as well as positive dogs for either *Bb*sl or *Ap*.

The medical relevance of the differences between the groups in the target parameters tick count at 48 h and positive status for *Bb*sl or *Ap* was quantified using the Mann–Whitney superiority measure (MW) and its two-sided 95.0% confidence interval as corresponding effect size.

The analysis was performed with the validated program Testimate Version 6.5 from IDV Gauting (IDV Data analysis & Study planning, Krailling, Germany).

### Results

#### Acaricidal efficacy

Tick counts are reported in Table 1; 100% efficacy was achieved in the 48-h counts using arithmetic mean (*p* = 0.0003) at 2 months after acaricidal treatment.

**Table 1:** Tick counts in study 1

Group	Dog ID	SD 65 (in situ counts 48 h p.i.)	SD 69 (6 days p.i.)
Non-treated group (group 1)	4945	20	14
	7537	21	10
	6531	16	13
	5933	19	7
	7472	22	10
	6891	16	11
	Arithm. mean	19.0	10.8
2mo-Seresto®-treated group (group 2)	6352, 5640, 7014, 6549, 7103, 4392, 5518, 7677	All dogs 0	All dogs 0
	Arithm. mean	0	0

*SD*, study day; *p.i.*, *post infestationem*

#### Tick testing for *Bb*sl and *Ap* in removed specimens

Individual analysis of the ticks removed from dogs on SD 69 showed that single ticks of the non-treated dogs tested positive for *Bb*sl or *Ap* by PCR.

In total, nine of the 65 removed ticks (from dog IDs 4945, 7537, 6531, 5933 and 7472) tested positive by PCR for *Bb*sl (13.8%).

In total, two of the 65 removed ticks (from dog IDs 6531 and 5933) were positive by PCR for *Ap* (3.1%).

#### Summary on *Bb*sI

Two dogs (IDs 4945, 6891) out of six of the non-treated dogs showed evidence of a *Bb*sl infection by being positive in all different test systems (skin biopsy PCR, skin biopsy culture, SNAP® 4Dx®, KELA/IBL) at different time points during the study, while none of the dogs of the treated group were positive. For details on time points and test systems, see Table 2.

**Table 2:** Summary on *Bb*sl test results of study 1

Group	Dog ID	SD 69 (6 days p.i.)		SD 76 (13 days p.i.)		SD 90 (27 days p.i.)				SD 104 (41 days p.i.)		SD 118 (55 days p.i.)					SD 132 (69 days p.i.)			SD 146 (83 days p.i.)					SD 175 (112 days p.i.)			SD 206 (143 days p.i.)			SD 230 (167 days p.i.)			SD 252 (189 days p.i.)			Combined *Bb*sl status (all tests and all SDs)
		KELA*	SNAP® 4Dx® (*Bb*sl)	KELA*	SNAP® 4Dx® (*Bb*sl)	KELA*	SNAP® 4Dx® (*Bb*sl)	Skin biopsy		KELA*	SNAP® 4Dx® (*Bb*sl)	KELA*	IBL**	SNAP® 4Dx® (*Bb*sl)	Skin biopsy		KELA*	IBL**	SNAP® 4Dx® (*Bb*sl)	KELA*	IBL**	SNAP® 4Dx® (*Bb*sl)	Skin biopsy		KELA*	IBL**	SNAP® 4Dx® (*Bb*sl)	KELA*	IBL**	SNAP® 4Dx® (*Bb*sl)	KELA*	IBL**	SNAP® 4Dx® (*Bb*sl)	KELA*	IBL**	SNAP® 4Dx® (*Bb*sl)	
								PCR	Culture						PCR	Culture							PCR	Culture													
Non-treated group (group 1)	4945	–	–	–	–	–	–	1 pos	1 pos	–	pos	116.6	pos	pos	–	–	150.1	pos	pos	252.3	pos	pos	2 pos	–	253.8	pos	pos	256.5	pos	pos	257.1	pos	pos	301.4	pos	pos	pos
	7537	–	–	–	–	–	–	–	–	–	–	–	–	–	–	–	–	n.d.	–	–	n.d.	–	–	–	–	–	–	–	n.d.	–	–	n.d.	–	–	n.d.	–	–
	6531	–	–	–	–	–	–	–	–	–	–	–	–	–	–	–	–	n.d.	–	–	n.d.	–	–	–	–	–	–	–	n.d.	–	–	n.d.	–	–	n.d.	–	–
	5933	–	–	–	–	–	–	–	–	–	–	–	–	–	–	–	–	n.d.	–	–	n.d.	–	–	–	–	–	–	–	n.d.	–	–	n.d.	–	–	n.d.	–	–
	7472	–	–	–	–	–	–	–	–	–	–	–	–	–	–	–	–	n.d.	–	–	n.d.	–	–	–	–	–	–	–	n.d.	–	–	n.d.	–	–	n.d.	–	–
	6891	–	–	–	–	–	–	–	1 pos	–	–	–	pos	–	1 pos	–	104.4	pos	–	–	n.d.	pos	1 pos	–	–	pos	pos	155.7	pos	pos	160.3	pos	pos	216.9	pos	pos	pos
2mo-Seresto®-treated group (group 2)	6352	–	–	–	–	–	–	–	–	–	–	–	–	–	–	–	–	n.d.	–	–	n.d.	–	–	–	–	–	–	–	n.d.	–	–	n.d.	–	–	n.d.	–	–
	5640	–	–	–	–	–	–	–	–	–	–	–	–	–	–	–	–	n.d.	–	–	n.d.	–	–	–	–	–	–	–	n.d.	–	–	n.d.	–	–	n.d.	–	–
	7014	–	–	–	–	–	–	–	–	–	–	125.0	–	–	–	–	193.5	–	–	–	n.d.	–	–	–	121.7	–	–	117.2	–	–	– ^a^	–	–	– ^a^	–	–	–
	6549	–	–	–	–	–	–	–	–	–	–	–	–	–	–	–	–	n.d.	–	–	n.d.	–	–	–	–	–	–	–	n.d.	–	–	n.d.	–	–	n.d.	–	–
	7103	–	–	–	–	–	–	–	–	–	–	–	–	–	–	–	–	n.d.	–	–	n.d.	–	–	–	–	–	–	–	n.d.	–	–	n.d.	–	–	n.d.	–	–
	4392	–	–	–	–	–	–	–	–	–	–	–	–	–	–	–	–	n.d.	–	–	n.d.	–	–	–	–	–	–	–	n.d.	–	–	n.d.	–	–	n.d.	–	–
	5518	–	–	–	–	–	–	–	–	–	–	–	–	–	–	–	–	n.d.	–	–	n.d.	–	–	–	–	–	–	–	n.d.	–	–	n.d.	–	–	n.d.	–	–
	7677	–	–	–	–	–	–	–	–	–	–	–	–	–	–	–	–	n.d.	–	–	n.d.	–	–	–	–	–	–	–	n.d.	–	–	n.d.	–	–	n.d.	–	–

*SD*, study day; *p.i.*, *post infestationem*; *n.d.*, not determined; *KELA (kinetic enzyme-linked immunosorbent assay) cut-off ≤ 100 units; **IBL, immunoblot; *−*, negative result; *pos*, positive result; *1 pos*, one positive skin sample of two samples in total; *2 pos*, two positive skin samples of two samples in total

^a^borderline positive KELA value when including standard deviation, thus IBL performed

#### Summary on *Ap*

Four dogs (IDs 4945, 6531, 5933, 7472) of the six non-treated dogs showed evidence of an *Ap* infection by being *Ap* positive in different test systems (two in the buffy coat PCR, one in the SNAP® 4Dx®, three in the skin biopsy PCR) during the study, while none of the dogs of the treated group were positive. For details on time points and test systems, see also Table 3.

**Table 3:** Summary on *Ap* test results of study 1

Group	Dog ID	SD 69 (6 days p.i.)		SD 76 (13 days p.i.)		SD 90 (27 days p.i.)			SD 104 (41 days p.i.)		SD 118 (55 days p.i.)			SD 132 (69 days p.i.)		SD 146 (83 days p.i.)			SD 175 (112 days p.i.)		SD 206 (143 days p.i.)		SD 230 (167 days p.i.)		SD 252 (189 days p.i.)		Combined *Ap* status (all tests and all SDs)
		Buffy coat PCR	SNAP® 4Dx® (*Ap*)	Buffy coat PCR	SNAP® 4Dx® (*Ap*)	Buffy coat PCR	SNAP® 4Dx® (*Ap*)	Skin biopsy PCR	Buffy coat PCR	SNAP® 4Dx® (*Ap*)	Buffy coat PCR	SNAP® 4Dx® (*Ap*)	Skin biopsy PCR	Buffy coat PCR	SNAP® 4Dx® (*Ap*)	Buffy coat PCR	SNAP® 4Dx® (*Ap*)	Skin biopsy PCR	Buffy coat PCR	SNAP® 4Dx® (*Ap*)	Buffy coat PCR	SNAP® 4Dx® (*Ap*)	Buffy coat PCR	SNAP® 4Dx® (*Ap*)	Buffy coat PCR	SNAP® 4Dx® (*Ap*)	
Non-treated group (group 1)	4945	–	–	–	–	–	–	–	–	–	–	–	–	–	–	–	–	1 pos	–	–	–	–	–	–	–	–	pos
	7537	–	–	–	–	–	–	–	–	–	–	–	–	–	–	–	–	–	–	–	–	–	–	–	–	–	–
	6531	–	–	–	–	–	–	–	–	–	–	–	–	–	–	–	–	1 pos	–	–	–	–	–	–	–	–	pos
	5933	–	–	–	–	pos	–	–	pos	pos	pos	pos	–	pos	pos	pos	pos	–	pos	pos	–	pos	–	pos	–	pos	pos
	7472	–	–	–	–	–	–	–	pos	–	–	–	–	–	–	–	–	1 pos	–	–	–	–	–	–	–	–	pos
	6891	–	–	–	–	–	–	–	–	–	–	–	–	–	–	–	–	–	–	–	–	–	–	–	–	–	–
2mo-Seresto®-treated group (group 2)	6352, 5640 7014, 6549 7103, 4392, 5518, 7677	All treated dogs were negative for *Ap* antibodies (based on SNAP® 4Dx®) and *Ap* DNA (in buffy coat and skin PCR) on all tested days																									All negative

*SD*, study day; *p.i.*, *post infestationem*; −, negative result; *pos*, positive result; *1 pos*, one positive skin sample of two samples in total

#### Pathogen blocking efficacy and overall status

Two (*Bb*sl) and four (*Ap*) of six non-treated control dogs were positive, whereas none of the dogs in the 2mo-Seresto®-treated group were positive. For *Bb*sl, no significant difference could be demonstrated (*p* = 0.167) due to the low number of animals testing positive in the non-treated control group. For *Ap*, the 2mo-Seresto®-treated group demonstrated significantly fewer animals with a positive status (none) as compared to the non-treated control group (*p* = 0.009).

#### Clinical signs, adverse events and safety evaluation

None of the infected dogs showed any local or systemic signs that might be classically attributed to Lyme borreliosis or anaplasmosis (Appel et al. 1993; Magnarelli et al. 1987; Cohen et al. 1990; Greig et al. 1996; Kohn et al. 2008).

No serious treatment-related adverse events were observed. Four of the treated dogs showed mild hair coat and skin changes (areas of alopecia at the neck in three dogs and an erythema in the dorsal neck area in one dog).

## Study 2 (USA)

### Materials and methods

#### Study group design

This study was a parallel group design, single centre, controlled, partially randomised, long-term efficacy study containing ten dogs per study group, conducted at the College of Veterinary Medicine, Auburn University, Auburn, USA. The study was approved by the Auburn University IACUC. Blinding was achieved by separation of function: persons that performed the post-treatment laboratory analysis were different from those that performed group allocation, treatment and sampling.

Thirty healthy male and female beagle dogs at least 17 months of age, with a body weight of 8.7 to 14.1 kg and confirmed negative for *Bb*sl- and *Ap*-specific antibodies (the same test systems used as for serological testing during the study, see below under “Laboratory procedures”) were used in the study. They were allocated into three groups of ten dogs as follows:

- Non-treated control group (group 1: *n* = 10)
- Seresto®-treated group 1 month prior to tick infestations (1mo-Seresto®, group 2: *n* = 10)
- Seresto®-treated group 7 months prior to tick infestations (7mo-Seresto®, group 3: *n* = 10)

Dogs in the 7-months Seresto®-treated group (group 3) had collars applied 6 months before study start (i.e. 7 months prior to tick infestation) and were thus included without randomisation. Dogs in the non-treated control group (group 1) and the 1-month Seresto®-treated group (group 2) were ranked according to body weight (highest to lowest) and randomly assigned to the two groups. Any ties were broken by animal ID, highest to lowest.

No history of ectoparasiticidal treatment was recorded for the dogs prior to collar application. Thorough clinical examinations including the following aspects were conducted in all dogs 11 days prior to tick infestation and in adapted form whenever an abnormal general health status event was observed: general appearance, rectal temperature, eyes, cardiovascular system, superficial lymph nodes, ears, respiratory system, oral cavity, abdomen palpation, faeces, genitourinary system (external genitalia, urine), skin/hair coat, behavioural attitude and locomotion/musculature. Additionally, daily health observations were performed throughout the study.

#### Dose and administration of the investigational veterinary product

The Seresto® collar was fitted according to label instructions to the dogs of the two treatment groups. As the day of treatment SD 0 was different for the two treated groups, group affiliation is subsequently added as subscript to the SDs reported, to better differentiate between the two treatment intervals (7 months _[7mo]_ and 1 month _[1mo]_ prior to tick infestation) and the non-treated control group (negative _[neg]_).

#### Tick infestation of dogs

Approximately 80 *I. scapularis* ticks (about 50:50 sex ratio), naturally infected with *Bb*sl and *Ap*, were released onto the dogs on SD_7mo_ 219/SD_neg+1mo_ 32 after collar application. The ticks used for infestation were collected from the wild by flag dragging in a known infested habitat in southern Rhode Island, USA. PCR detected 54% *Bb*sl-positive ticks and 12% *Ap*-positive ticks in a representative sample of 30 ticks (15 females and 15 males) of the habitat. The PCR for *Bb*sl and *Ap* detection was conducted at the Department of Plant Sciences and Entomology, College of Environmental Sciences, University of Rhode Island, USA. In detail, DNA extraction of the field-collected ticks was performed using DNeasy® Blood and Tissue Kit (QIAGEN Inc., Valencia, CA, USA) in a modified protocol as described in McCall et al. (2011). To monitor the presence of *Bb*sl and *Ap* DNA in the ticks, DNA was analysed by real-time PCR using the following primer sequences: [A2 (5′-GTT TTG TAA TTT CAA CTG CTG ACC-3′) and A4 (5′-CTG CAG CTT GGA ATT CAG GCA CTT C-3′)] (Nocton et al. 1994) for *B. burgdorferi* sensu stricto PCR, [ge3a (5′-CAC ATG CAA GTC GAA CGG ATT ATT C-3′) and ge10 (5′-TTC CGT TAA GAA GGA TCT AAT CTC C-3′)] and [ge2 (5′-GGC AGT ATT AAA AGC AGC TCC AGG-3′) and ge9 (5′-AAC GGA TTA TTC TTT ATA GCT TGC T-3′)] (Massung et al. 1998) for primary and nested *Ap* PCR. Detailed PCR conditions are listed in McCall et al. (2011) as well as in Nocton et al. (1994), Massung et al. (1998) and Massung and Slater (2003).

#### On animal procedures

##### Tick counting

As in study 1, in situ tick thumb counts by intensive examination and palpation of all body parts were carried out on all dogs 48 h after infestation (SD_neg+1mo_ 34/SD_7mo_ 221) for acaricidal efficacy according to Marchiondo et al. (2013). Any non-attached live ticks were not removed but counted and included in the thumb counts. To enhance the potential of pathogen transmission, ticks were removed 5 days (SD_neg+1mo_ 37/SD_7mo_ 224) after infestation when they were again counted and categorised, both according to the W.A.A.V.P. guideline (Marchiondo et al. 2013).

##### Blood sampling

**Fig. 2:**
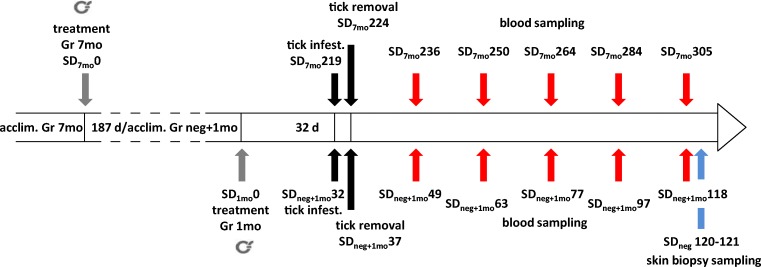
Key study dates of study 2. SD, study day; Gr, group; d, days; , collar treatment; acclim., acclimatisation. SD 0 is generally considered the day of treatment; group affiliation is added as subscript to SD to better differentiate between the two different treatment intervals (7 months _[7mo]_ and 1 month _[1mo]_ prior to tick infestation) and the non-treated control group (negative _[neg]_); the SDs for blood sampling correspond to 17, 31, 45, 65 and 86 days post tick infestation in all study groups

Blood sampling for serum collection was performed on all dogs on 17, 31, 45, 65 and 86 days post-tick infestation (i.e. SDs_neg+1mo_/SDs_7mo_ 49/236, 63/250, 77/264, 97/284, and 118/305; see Fig. 2 for details). Samples were stored at − 80 °C until analysis.

##### Skin biopsy sampling

Four skin biopsy samples were collected in the control group according to the protocol below following the schedule in Fig. 2. For biopsy sampling, dogs were sedated using 375 μg dexmedetomidine/kg body weight intravenously. Hair was clipped at the biopsy site. The site was disinfected using chlorhexidine scrub, followed by alcohol. To block the biopsy site locally, 0.5–1.0 ml lidocaine 2% was injected subcutaneously below the sampling site, where necessary. Biopsies were collected with a commercially available 4-mm-diameter punch, and skin was closed using 3–0 PDS (polydioxanone suture) in 1–2 interrupted sutures. Finally, sedation was reversed using atipamezole intramuscularly in the amount corresponding to the volume of dexmedetomidine used. Sutures were removed 10–14 days later.

As no attached ticks were found in the 7mo- and 1mo-Seresto®-treated groups, no skin biopsy samples were taken in those groups. In the untreated control dogs, biopsies were taken from the area of known tick attachment sites. Biopsy samples were analysed at the Animal Health Diagnostic Centre at Cornell University as described below in “Molecular screening (*Bb*sl in skin biopsies)”.

#### Laboratory procedures

##### Serological screening (*Bb*sl)

The same general approach for serological testing as in study 1 was used. Again, the automated KELA (Shin et al. 1993; Barth et al. 2014) was combined with the immunoblot (stated as IBL) (Borrelia Veterinär plus OspA LINE; Sekisui Virotech GmbH, Rüsselsheim, Germany) as the second step. In contrast to study 1 and in order to enhance accuracy, both tests were performed for all samples, independent of the KELA value. Dogs with a positive IBL were considered positive and dogs with a negative IBL result were considered to be negative for *Bb*sl antibodies, irrespective of a positive or equivocal KELA result. All tests were performed at the Institute for Infectious Diseases and Zoonoses, Department of Veterinary Sciences, Faculty of Veterinary Medicine, LMU Munich, Germany.

Additionally, a commercial immunoassay SNAP® 4Dx® Plus (IDEXX Laboratories, Inc., Westbrook, ME, USA) was used (performed at the Department of Pathobiology, College of Veterinary Medicine, Auburn University, USA).

Interpretation of the serological results including all three test systems was in accordance with study 1.

##### Molecular screening (*Bb*sl in skin biopsies)

DNA extraction of the skin biopsy samples and a subsequent duplex PCR were performed according to the protocol described by Pahl et al. (1999) [forw (5′-TCT TTT CTC TGG TGA GGG AGC T-3′), rev (5′-TCC TTC CTG TTG AAC ACC CTC T-3′)]. Testing was conducted at the Animal Health Diagnostic Centre, Cornell University, USA.

##### Serological screening (*Ap*)

Serum was examined using the SNAP® 4Dx® Plus. For SDs on which the test was performed, see Fig. 2. Samples that were positive using SNAP® 4Dx® Plus were considered to be positive for *Ap*, disregarding any potential cross reactivity for other *Anaplasma* species, as the tick batch used for infection were confirmed to be *Ap* positive by PCR. Testing was performed at the Department of Pathobiology, College of Veterinary Medicine, Auburn University, USA.

#### Evaluation of efficacy

##### Acaricidal efficacy

Acaricidal efficacy was calculated according to Marchiondo et al. (2013) as described for study 1.

##### Pathogen blocking efficacy

The percentage blocking efficacies for the treatment groups were calculated as described for study 1.

#### Statistical analysis

Since the study design did not allow for randomisation of dogs in the 7mo-Seresto®-treated group (group 3), only descriptive summaries are provided for this group. All statistical analyses were performed on the 1mo-Seresto®-treated group (group 2) and the non-treated control group (group 1) data.

The Wilcoxon rank test was used to evaluate the acaricidal efficacy by analysing the tick counts on SD_neg+1mo_ 34. Binary categories (positive or non-positive) were assigned to each numeric serology results and analysed using Fisher’s exact test. For *Ap*, the proportions (number of positives/total number) of SNAP® 4Dx® Plus results were calculated for both groups on each study day and analysed using Fisher’s exact test. For *Bb*sl, combined results from three parameters (skin biopsy, SNAP® 4Dx® Plus and KELA/IBL) provided an overall assessment (positive or negative) for each animal. These results were analysed using Fisher’s exact test.

All computations were performed using SAS version 9 (SAS® Institute, Cary, NC, USA). The significance level was 0.05.

### Results

#### Acaricidal efficacy

Tick counts are reported in Table 4; 100% efficacy was achieved in the 48-h counts for both the 1mo- (*p* < 0.0001) and the 7mo-Seresto® group, using arithmetic means. No statistical calculation was performed for the 7mo-Seresto® group as those animals were not randomised with the rest of the study animals.

**Table 4:** Tick counts in study 2

Group	Dog ID	SD_neg+1mo_ 34/SD_7mo_ 221 (in situ counts 48 h p.i.)	SD_neg+1mo_ 37/SD_7mo_ 224 (5 days p.i.)
Non-treated group (group 1)	TJI-2	14	32
	ISK-2	11	25
	VPI-2	4	38
	TII-2	25	46
	EXJ-2	11	34
	KTL-2	12	33
	YOO-2	15	34
	AFO-2	17	38
	WVO-2	18	39
	KIP-2	8	29
	Arithm. mean	13.5	34.8
1mo-Seresto®-treated group (group 2)	UBL-2, VOI-2, BHP-2, WJP-2, ETJ-2, QVJ-2, GIJ-2, GYK-2, IUK-2, ZFP-2	All dogs 0	All dogs 0
	Arithm. mean	0	0
7mo-Seresto®-treated group (group 3)	XQP-2, ZYG-2, GAG-2, OYJ-2, TVQ-2, VEI-2, OIJ-2, PIJ-2, BJH-2, ITK-2	All dogs 0	All dogs 0
	Arithm. mean	0	0

*SD*, study day; *p.i.*, *post infestationem*

#### Summary on *Bb*sI

Generally, a dog was evaluated positive, when it was positive in one of the applied test systems (either KELA/IBL or SNAP or skin biopsy). All ten dogs in the non-treated control group showed evidence of a *Bb*sl infection by being positive in different test system combinations (seven of ten dogs in all test systems; ten of ten in the KELA/IBL combination; all but one in the SNAP® 4Dx® Plus; eight of ten in the skin biopsy PCR) during the study, while all dogs in both treatment groups stayed negative for *Bb*sl evidence. For details, see also Table 5.

**Table 5:** Summary on *Bb*sl test results of study 2

Group	Dog ID	SD_neg+1mo_ 49/SD_7mo_ 236 (17 days p.i.)			SD_neg+1mo_ 63/SD_7mo_ 250 (31 days p.i.)			SD_neg+1mo_ 77/SD_7mo_ 264 (45 days p.i.)			SD_neg+1mo_ 97/SD_7mo_ 284 (65 days p.i.)			SD_neg+1mo_ 118/SD_7mo_ 305 (86 days p.i.)			SD_neg_ 120–121 (88–89 days p.i.)	Combined *Bb*sl status (all tests and all SDs)
		KELA*	IBL**	SNAP® 4Dx® Plus (*Bb*sl)	KELA*	IBL**	SNAP® 4Dx® Plus (*Bb*sl)	KELA*	IBL**	SNAP® 4Dx® Plus (*Bb*sl)	KELA*	IBL**	SNAP® 4Dx® Plus (*Bb*sl)	KELA*	IBL**	SNAP® 4Dx® Plus (*Bb*sl)	Skin biopsy PCR	
Non-treated group (group 1)	TJI-2	−	−	−	182.9	pos	−	247.7	pos	−	235.7	pos	−	256.2	pos	−	4 pos	pos
	ISK-2	−	−	−	189.5	pos	pos	181.8	pos	pos	376.9	pos	pos	314.3	pos	pos	3 pos, 1?	pos
	VPI-2	110.2	pos	−	130.3	pos	−	189.4	pos	pos	489.2	pos	pos	390.4	pos	pos	4 pos	pos
	TII-2	−	−	−	−	pos	−	122.3	pos	−	234.2	pos	pos	219.5	pos	pos	1 pos, 1?	pos
	EXJ-2	228.9	−	−	256.1	pos	−	178.4	pos	−	123.7	pos	−	109.8	pos	pos	1?	pos
	KTL-2	123.4	pos	−	109.9	pos	pos	155.9	pos	pos	328.2	pos	pos	268.1	pos	pos	4 pos	pos
	YOO-2	387.8	−	−	299.7	pos	−	134.4	pos	pos	169.5	pos	pos	126.2	pos	pos	−	pos
	AFO-2	114.2	−	−	205.6	pos	−	155.8	pos	pos	250.9	pos	pos	393.4	pos	pos	4 pos	pos
	WVO-2	−	−	−	166.5	pos	−	150.6	pos	pos	338.8	pos	pos	302.7	pos	pos	2 pos	pos
	KIP-2	120.3	−	−	124.1	pos	−	114.0	pos	−	110.3	pos	−	−	pos	pos	2 pos	pos
1mo-Seresto®-treated group (group 2)	UBL-2	−	−	−	−	−	−	−	−	−	−	−	−	−	−	−	n.d.	−
	VOI-2	−	−	−	−	−	−	−	−	−	−	−	−	−	−	−	n.d.	−
	BHP-2	156.1	−	−	125.2	−	−	−	−	−	−	−	−	113.9	−	−	n.d.	−
	WJP-2	−	−	−	−	−	−	−	−	−	−	−	−	−	−	−	n.d.	−
	ETJ-2	−	−	−	−	−	−	−	−	−	−	−	−	−	−	−	n.d.	−
	QVJ-2	−	−	−	−	−	−	−	−	−	−	−	−	−	−	−	n.d.	−
	GIJ-2	−	−	−	−	−	−	−	−	−	−	−	−	−	−	−	n.d.	−
	GYK-2	−	−	−	−	−	−	−	−	−	−	−	−	−	−	−	n.d.	−
	IUK-2	−	−	−	−	−	−	−	−	−	−	−	−	−	−	−	n.d.	−
	ZFP-2	−	−	−	−	−	−	−	−	−	−	−	−	−	−	−	n.d.	−
7mo-Seresto®-treated group (group 3)	XQP-2	−	−	−	−	−	−	−	−	−	−	−	−	−	−	−	n.d.	−
	ZYG-2	−	−	−	−	−	−	−	−	−	−	−	−	−	−	−	n.d.	−
	GAG-2	−	−	−	−	−	−	−	−	−	−	−	−	−	−	−	n.d.	−
	OYJ-2	−	−	−	−	−	−	−	−	−	−	−	−	−	−	−	n.d.	−
	TVQ-2	−	−	−	−	−	−	−	−	−	−	−	−	−	−	−	n.d.	−
	VEI-2	−	−	−	−	−	−	−	−	−	−	−	−	−	−	−	n.d.	−
	OIJ-2	−	−	−	−	−	−	−	−	−	−	−	−	−	−	−	n.d.	−
	PIJ-2	−	−	−	−	−	−	−	−	−	−	−	−	−	−	−	n.d.	−
	BJH-2	104.7	−	−	109.8	−	−	165.7	−	−	128.5	−	−	121.1	−	−	n.d.	−
	ITK-2	−	−	−	−	−	−	−	−	−	−	−	−	−	−	−	n.d.	−

*SD*, study day; *p.i.*, *post infestationem*; *n.d.*, not determined; *KELA (kinetic enzyme-linked immunosorbent assay) with cut-off ≤ 100 units; **IBL, immunoblot; *−*, negative result; *pos*, positive result; *1–4 pos*, one to four positive skin sample of four samples in total; *1?*, one questionable positive skin sample of four samples in total

#### Summary on *Ap*

All ten dogs in the non-treated control group showed evidence of an *Ap* infection by being *Ap* positive in the test system (SNAP® 4Dx® Plus) during the course of the study, latest 45 days p.i., while all dogs in both treatment groups remained negative for *Ap*. For details, see also Table 6.

**Table 6:** Summary on *Ap* test results of study 2

Group	Dog ID	SD_neg+1mo_ 49/SD_7mo_ 236 (17 days p.i.)	SD_neg+1mo_ 63/SD_7mo_ 250 (31 days p.i.)	SD_neg+1mo_ 77/SD_7mo_ 264 (45 days p.i.)	SD_neg+1mo_ 97/SD_7mo_ 284 (65 days p.i.)	SD_neg+1mo_ 118/SD_7mo_ 305 (86 days p.i.)
		SNAP® 4Dx® Plus (*Ap*)	SNAP® 4Dx® Plus (*Ap*)	SNAP® 4Dx® Plus (*Ap*)	SNAP® 4Dx® Plus (*Ap*)	SNAP® 4Dx® Plus (*Ap*)
Non-treated group (group 1)	TJI-2	−	−	pos	pos	pos
	ISK-2	−	pos	pos	pos	pos
	VPI-2	−	pos	pos	pos	pos
	TII-2	−	pos	pos	pos	pos
	EXJ-2	−	pos	pos	pos	pos
	KTL-2	−	pos	pos	pos	pos
	YOO-2	−	pos	pos	pos	pos
	AFO-2	−	pos	pos	pos	pos
	WVO-2	−	pos	pos	pos	pos
	KIP-2	−	pos	pos	pos	pos
1mo-Seresto®-treated group (group 2)	UBL-2, VOI-2, BHP-2, WJP-2, ETJ-2, QVJ-2, GIJ-2, GYK-2, IUK-2, ZFP-2	All treated dogs were negative for *Ap* antibodies (based on SNAP® 4Dx® Plus) on all tested days				
7mo-Seresto®-treated group (group 3)	XQP-2, ZYG-2, GAG-2, OYJ-2, TVQ-2, VEI-2, OIJ-2, PIJ-2, BJH-2, ITK-2	All treated dogs were negative for *Ap* antibodies (based on SNAP® 4Dx® Plus) on all tested days				

*SD*, study day; *p.i.*, *post infestationem*; *−*, negative result; *pos*, positive result

#### Pathogen blocking efficacy and overall status

All non-treated animals turned positive for both pathogens (*Bb*sl and *Ap*), whereas all dogs in the 1mo- (*p* < 0.05) and the 7mo-Seresto®-treated group remained negative.

Significance was not calculated for the 7mo-Seresto®-treated group as these animals were not randomised with the other study animals.

#### Clinical signs, adverse events and safety evaluation

None of the infected dogs showed any local or systemic signs that might be classically attributed to Lyme borreliosis or ana-plasmosis (Appel et al. 1993; Magnarelli et al. 1987; Cohen et al. 1990; Greig et al. 1996; Kohn et al. 2008).

No serious treatment-related adverse events were observed. Eight dogs in the 7mo-Seresto®-treated group showed mild signs of hair loss and skin irritation on the neck. Mild signs of hair loss were also reported in one dog in the 1mo-Seresto®-treated group.

## Discussion

In recent years, the efficacy of ectoparasiticides has increasingly been judged not only for its acaricidal effects, but additionally for its ability to prevent pathogen transmission.

Concerning the pathogens in focus of the reported studies, *Bb*sl and *Ap*, various actives and formulations have been tested regarding their capacity to prevent microorganism transmission from *I. scapularis* to dogs (Baker et al. 2016; Blagburn et al. 2004; Elfassy et al. 2001; Honsberger et al. 2016; Hunter et al. 2002; McCall et al. 2011; Spencer et al. 2003), but according to the authors’ knowledge, one of the reported studies is the first to test the prevention of pathogen transmission for up to 7 months after a single treatment (collar application).

Study 1 was designed to evaluate Seresto®’s protective capacity on tick-borne agent transmission at 2 months following collar application. Protection against infection with *Bb*sl was 100% with none of the treated dogs testing positive. However, due to the low number of *Bb*sl-infected dogs in the non-treated control group (two of six), superiority for the treatment group versus the non-treated control group (0.00 vs. 33.33%) could not be proven (*p* = 0.167). In the representative batch testing of the collected ticks, the infection rate of *Bb*sl in study 1 ranged between 19.8 and 33%. This is comparable to data in field studies in Germany with infection rates in ticks varying from 9.5 to 34.1% (Bingsohn et al. 2013; Schreiber et al. 2014; Tappe et al. 2014; May et al. 2015). However, retrospective PCR testing of the attached ticks only proved 13.8% to be *Bb*sl infected. Thus, a potential reason for the low number of *Bb*sl-positive dogs in the non-treated control group in study 1 could be an insufficient number of borrelial organisms carried by those ticks that actually fed on the study animals. Additionally, European ticks carry a mixture of *Borrelia* species with potentially different infectivity for the canine host. This could also be a reason for a lower *Bb*sl infection rate in the dogs in study 1 using the European ticks. Furthermore, a discrepancy between attached ticks screened positive and a negative serological status of the dogs and vice versa could be observed, meaning attached ticks were positive for *Bb*sl, but the corresponding canine host did not develop any antibodies or the dog was serologically positive, but no positive tick was detected during PCR. Here again, the above-mentioned factors of infection load within the tick, *Borrelia* species and also time of attachment may play a role for this deviation.

In the same study (study 1), 100% protection against infection with *Ap* was achieved at the 2 months after treatment time point, with none of the treated dogs positive, while four of six dogs in the non-treated control group were positive for *Ap-*specific DNA. Superiority of the treated group versus the non-treated control group (0.00 vs. 66.67%) was significant (*p* = 0.009). *Ap* infection rates in field-collected ticks from Germany used in this study ranged between 2 and 5%, which generally is comparable to other field prevalence data from the country ranging from 2.1 to 6% in adult ticks (May and Strube 2014; Schicht et al. 2011; Hildebrandt et al. 2010). Thus, the *Ap* infection rate of 3.1% detected by PCR in the ticks removed from infested dogs during the study can be regarded as representative for field conditions. But as discussed for the *Bb*sl results, again attached *Ap-*positive ticks were detected in dogs which did not seroconvert and vice versa. Repeatedly, the infection load within the tick, the time of tick attachment and also the reduced number of attached ticks, which were screened (*n* =65 from a total of 180 female ticks on the six control dogs, which potentially got attached) might explain this discrepancy.

Based on the promising though not fully conclusive pathogen blocking results for *Bb*sl and *Ap* from the first study, the second study in the USA was conducted looking at two additional time points, 1 and 7 months after Seresto® collar application. Study 2 showed far higher infection rates in the non-treated animals (ten of ten for *Bb*sl, ten of ten for *Ap*) than study 1, which corresponds with the far higher infection rates found in the study ticks from the USA compared to the study ticks from Germany (*Bb*sl infection rate, 54% [USA] vs. up to 33% [Germany]; *Ap* infection rate, 12% [USA] vs. up to 5% [Germany]). Field data for *B. burgdorferi* in *I. scapularis* from the USA ranges from 7 to 52.1% (Levine et al. 2017; Serra et al. 2013; Schulze et al. 2013; Hamer et al. 2014). For *Ap*, field data from collected ticks in the USA ranges from 3.7 to 20% (Hamer et al. 2014; Prusinski et al. 2014; Roellig and Fang 2012).

Complete (100%) blocking efficacy was achieved for *Bb*sl and *Ap* for both evaluation time points (1 and 7 months after Seresto® collar application). Though results regarding transmission blocking were identical (no *Bb*sl- or *Ap-*positive dogs), superiority was calculated only for the 1-month Seresto®-treated group, as dogs in the 7-months group were pre-included without randomisation.

The ability of the Seresto® collar to prevent transmission of tick-borne pathogens to dogs has already been demonstrated—in laboratory as well as field studies—against a number of pathogens, including *Babesia canis* (Fourie et al. 2013b, 2017), *Babesia vogeli*, *A. platys* (Dantas-Torres et al. 2013) and *E. canis*-positive *Rhipicephalus sanguineus* ticks (Stanneck and Fourie 2013).

In both studies, the Seresto® collar was well tolerated by the dogs with only slight alopecia and some local skin irritation in some dogs. An efficacy of 100% for tick kill at 48 h was recorded for both tick species (*I. ricinus* and *I. scapularis*) for 1, 2 and 7 months after the placement of the collar, in line with the data from former studies (Stanneck et al. 2012b). In endemic areas, the use of acaricidal products within a tick-control program is strongly advised for the protection of both canine and public health. Due to its long-term efficacy, the Seresto® collar facilitates owner compliance and can be seen as a valuable tool in such a program.

## Conclusions

The Seresto® collar was tested for its ability to prevent transmission of *Bb*sl or *Ap* from *I. ricinus* at 2 months and from *I. scapularis* ticks at 1 and 7 months after application. Acaricidal efficacy as well as pathogen transmission blocking for *Bb*sl or *Ap* was shown to be 100% for all time points evaluated.

## Abbreviations

*Ap*: *Anaplasma phagocytophilum*
*Bb*sl: *Borrelia burgdorferi* sensu lato
CVBD: canine vector-borne disease
DNA: deoxyribonucleic acid
GCP: Good Clinical Practice
IACUC: Institutional Animal Care and Use Committee
IBL: immunoblot
ID: identity
i.e.: *id**est* (that is to say)
KELA: kinetic enzyme-linked immunosorbent assay
LANUV: [Landesamt für Natur, Umwelt und Verbraucherschutz] State Office for Nature, Environment and Consumer Protection
p.i.: *post infestationem* (after infestation)
PCR: polymerase chain reaction
SD: study day
vs.: versus
W.A.A.V.P.: World Association for the Advancement of Veterinary Parasitology
